# A Complex Case of Histoplasmosis in an Immunocompromised Patient: Diagnostic Challenges, Multidisciplinary Collaboration, and Environmental Factors

**DOI:** 10.7759/cureus.51276

**Published:** 2023-12-29

**Authors:** Samuel Sabzanov, Marc Ganz, Benjamin Mishail, Daniel Yusupov, Paul Fried, Menachem Jacobs, Daniel Miller

**Affiliations:** 1 Public Health Sciences, State University of New York Downstate Health Sciences University, New York, USA; 2 Internal Medicine, University Hospital of Brooklyn, New York, USA; 3 Internal Medicine, State University of New York Downstate Health Sciences University, New York, USA; 4 Internal Medicine, Icahn School of Medicine at Mount Sinai, Queens Hospital Center, New York, USA

**Keywords:** fungal infection, multidisciplinary, diagnosis, immunocompromised, histoplasmosis

## Abstract

Histoplasmosis is a systemic fungal infection caused by *Histoplasma capsulatum*, known for its protean clinical manifestations that often pose diagnostic challenges. Immunocompromised patients, such as those on immunosuppressive therapies or with HIV/AIDS, are particularly susceptible to severe forms of the disease. We present a case of a 55-year-old female with a complex medical history, including a renal transplant, who developed fever, malaise, nausea, and vomiting after a month-long stay in Panama. The patient's history included exposure to a bird with apparent infection and mold in her home. Her clinical presentation featured acute kidney injury, elevated liver enzymes, acalculous cholecystitis, and lung nodules. This intricate constellation of symptoms underscores the diverse nature of histoplasmosis presentations and its potential to mimic other diseases. The patient underwent a stepwise diagnostic approach involving imaging, microbiological tests, and multidisciplinary consultations. The positive Fungitell assay, *Histoplasma capsulatum* detection in urine, and identification of scattered subcentimeter lung nodules confirmed the diagnosis. This case underscores the significance of considering endemic areas, environmental exposures, and atypical clinical features in immunocompromised patients. The multidisciplinary approach facilitated appropriate management and treatment initiation with liposomal amphotericin B, highlighting the importance of collaboration among various medical specialties in complex cases. As such, this case report emphasizes the complexity of diagnosing and managing histoplasmosis in immunocompromised individuals and highlights the need for a comprehensive evaluation of atypical presentations.

## Introduction

Histoplasmosis is a systemic fungal infection caused by the dimorphic fungus *Histoplasma capsulatum*. It is known to have a diverse range of clinical presentations, often challenging diagnostic efforts due to its protean manifestations. The disease is endemic in certain geographical regions, including the Americas, with variations in prevalence and clinical patterns across different locales. Although histoplasmosis can affect individuals with various immune statuses, it is particularly noteworthy in immunocompromised patients, such as those with HIV/AIDS, organ transplant recipients, and patients on immunosuppressive therapies [1,2].

The standard presentation of histoplasmosis can encompass a spectrum of clinical features. The infection may range from asymptomatic or mild self-limited disease to severe disseminated forms. The respiratory route is the primary mode of infection, with the fungus often found in soil enriched with bird and bat droppings. Acute pulmonary histoplasmosis typically presents with influenza-like symptoms, including fever, cough, myalgias, and fatigue. This can sometimes lead to diagnostic confusion, especially during outbreaks or in regions where the disease is not endemic [3,4].

As histoplasmosis disseminates, it can affect various organ systems, leading to a myriad of manifestations. Disseminated histoplasmosis may involve the liver, spleen, bone marrow, and mucocutaneous tissues. It can also cause fever, weight loss, hepatosplenomegaly, lymphadenopathy, and pancytopenia, further complicating the diagnostic process [5,6]. The presentation of histoplasmosis can vary significantly based on the patient's immune status and underlying comorbidities, necessitating a high index of suspicion and a comprehensive diagnostic workup.

In this case report, we present an intriguing case of histoplasmosis that highlights the diagnostic challenges and complexities associated with this fungal infection. This case underscores the importance of considering histoplasmosis in the differential diagnosis of patients with atypical presentations, particularly with exposures to areas where the disease is endemic.

## Case presentation

The patient is a 55-year-old female with a complex medical history. Her past medical history includes hypertension, gastroesophageal reflux disease (GERD), a previous gastric sleeve surgery, hyperlipidemia, migraines, and a deceased donor renal transplant 13 years prior. She arrived at the ED with complaints of generalized malaise, intermittent fevers lasting for two weeks, and accompanying nausea and vomiting. Notably, she had spent the past month in Panama before presenting to the ED and admitted to exposure to mold in a home as well as a bird with an apparent skin feather infection. Her baseline creatinine was 1.6 mg/dl, which on presentation was elevated to a level of 3.2 mg/dl. Additionally, she had elevated liver enzymes with an aspartate aminotransferase of 266 U/L (normal 12-37 U/L), an alanine aminotransferase of 305 U/L (normal 15-65 U/L), an alkaline phosphatase of 399 U/L (normal 50-136 U/L), bilirubin of 1.5 mg/dl (normal 0.2-1.0 mg/dl), and a gamma-glutamyltransferase of 419 U/L (normal 0-50 U/L). Her temperature on admission was 101.5°F, and she displayed signs of sepsis. On physical examination, she exhibited tenderness to palpation in the lower left and right quadrants of her abdomen, along with a positive Murphy's sign. Given her history of renal transplant and immunosuppressive therapy including tacrolimus, mycophenolate, and prednisone, the patient was admitted to the transplant service for further evaluation due to her fevers, acute kidney injury (AKI), and elevated liver function tests.

A CT scan of the abdomen was conducted initially, revealing possible sludge in a non-distended gallbladder. Subsequent abdominal ultrasounds (US) did not indicate cholelithiasis or signs of acute cholecystitis. Infectious disease was consulted due to the suspicion of an infectious etiology for the acalculous cholecystitis and hepatitis, considering her immunosuppression and recent travel to Panama. The patient was started on Zosyn for treatment of acalculous cholecystitis. Given the persistence of symptoms, a percutaneous cholecystostomy drain was performed to address the acalculous cholecystitis. However, the patient remained febrile post-procedure. Micafungin was added to her treatment regimen due to concerns about potential fungal infections.

At this time, blood cultures taken on admission were negative, and urine cultures were negative. Hepatitis A, B, and C serology showed no evidence of infection. Epstein-Barr virus, *Cytomegalovirus*, *Legionella*, cryptococcal, Syphilis rapid plasma reagin, and a full respiratory viral panel did not reveal any positive results either.

The patient's urine was positive for BK virus, and a Fungitell assay was positive, indicating a potential fungal infection. Tests for dengue, chikungunya, zika, and histoplasma were sent.

**Figure 1:**
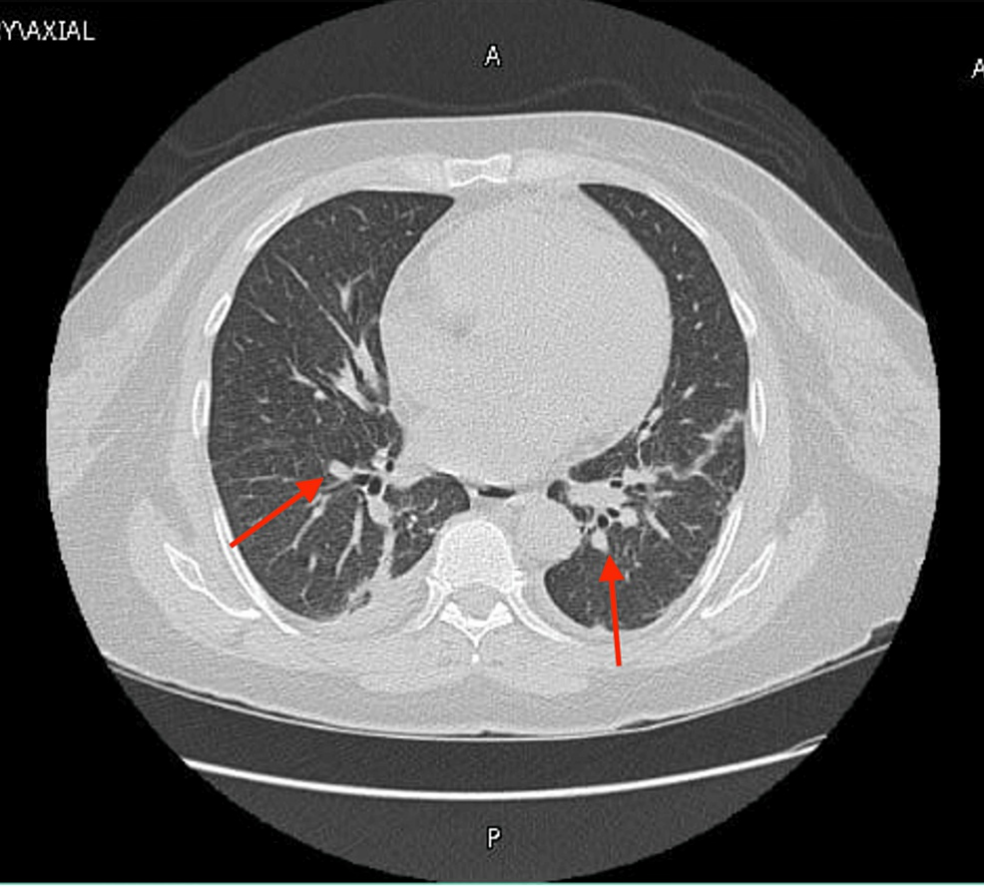
CT chest revealing multiple subcentimeter nodules Red arrows point to some of the nodules

The patient began to complain of shortness of breath on exertion, cough, and clear sputum. As a result, the patient was started on voriconazole, an antifungal medication used to cover potential aspergillosis infections. Further diagnostic investigations included a CT scan of the chest, which revealed scattered subcentimeter nodules throughout both lungs (Figure 1).

One week later, the patient developed new-onset headaches and pain associated with extraocular movements. She also reported painful and bleeding ulcers in the perineal region since starting Zosyn. Given these symptoms, brain MRI, ophthalmology, and gynecology consults were ordered. Pulmonary was additionally consulted for a potential bronchoalveolar lavage (BAL). The patient was scheduled for endobronchial US and BAL. Due to the evolving clinical picture and the need for a more comprehensive assessment, the patient was transferred from the surgical service to the medical service.

Soon after being transferred, the patient's pending labs showed that her urine histoplasma antigen was positive, which was then thought to be the explanation for her symptoms. In light of this finding, the recommendation was made to discontinue voriconazole and micafungin and to start liposomal amphotericin B. This led to clinical improvement, and the remaining tests and consultations were ultimately cancelled.

## Discussion

The patient's medical history, including a deceased donor renal transplant and ongoing immunosuppressive therapy, significantly contributed to her susceptibility to opportunistic infections like histoplasmosis. Immunocompromised patients, such as those on immunosuppressive medications or with HIV/AIDS, are at increased risk of severe and disseminated histoplasmosis due to impaired cellular immunity [7,8]. The patient's recent travel to Panama, an area known for histoplasmosis endemicity, further heightened the suspicion of a fungal infection. This scenario underscores the importance of considering travel history when evaluating patients with febrile illnesses, as exposure to endemic regions can significantly impact the differential diagnosis [9].

Furthermore, the patient's exposure to a bird with an apparent infection and to mold adds a crucial layer to the diagnostic considerations. Birds, particularly those in environments rich in bird and bat droppings, are recognized as sources of *Histoplasma capsulatum*, given its presence in soil enriched with such organic matter [3]. The exposure to mold, often found in areas with high humidity and organic materials, further raises the possibility of inhalation of fungal spores, thus increasing the potential for fungal infection. These environmental exposures emphasize the need to consider not only clinical factors but also the patient's history of interactions with potential sources of infection, particularly in cases with complex and atypical presentations.

The patient's clinical presentation was intricate and involved multiple organ systems. Initial symptoms, including generalized malaise, intermittent fevers, nausea, vomiting, and AKI, posed a diagnostic challenge. The elevated liver enzymes, acalculous cholecystitis, and subsequent development of ulcers in the perineal region were atypical and initially perplexing manifestations. These varied clinical features exemplify the protean nature of histoplasmosis and its propensity to mimic other diseases, often leading to diagnostic delay or confusion [2,10].

The stepwise approach taken in this case to reach a definitive diagnosis is noteworthy. Given the patient's immunosuppressed state, broad-spectrum antibiotics were initially administered due to suspicion of bacterial infection. The initiation of antifungal therapy, such as voriconazole, was reasonable considering the evolving clinical presentation and immunocompromised status. However, the positive Fungitell assay, indicating fungal infection, and the subsequent identification of *Histoplasma capsulatum* in the urine led to a pivotal turning point in the diagnosis. This highlights the utility of fungal biomarkers and diagnostic assays in guiding treatment decisions, especially in the absence of immediate positive culture results [11].

The case also underscores the need for a multidisciplinary approach in complex cases. The involvement of infectious disease, pulmonary, gynecology, and ophthalmology services facilitated a comprehensive evaluation of the patient's symptoms. The consideration of differentials such as aspergillosis due to lung nodules further illustrates the challenges of distinguishing between different fungal infections with overlapping clinical features.

## Conclusions

The presented case serves as a stark reminder of the intricacies and challenges that can arise when diagnosing and managing histoplasmosis in immunocompromised individuals. The patient's unique medical history, recent travel, and environmental exposures all played pivotal roles in shaping her complex clinical presentation. This case underscores the critical importance of maintaining a high level of suspicion for histoplasmosis in patients with atypical symptoms, a history of exposure to endemic areas, and compromised immune systems.

Moreover, the diagnostic journey in this case showcases the value of fungal biomarkers and diagnostic assays, which can guide treatment decisions when cultures do not provide immediate answers. The multidisciplinary approach taken in this patient's care emphasizes the need for collaboration among various medical specialties when dealing with complex and challenging cases, especially when differential diagnoses include multiple fungal infections with overlapping clinical features.
